# Molecular Characterization of Podoviral Bacteriophages Virulent for *Clostridium perfringens* and Their Comparison with Members of the *Picovirinae*

**DOI:** 10.1371/journal.pone.0038283

**Published:** 2012-05-29

**Authors:** Nikolay V. Volozhantsev, Brian B. Oakley, Cesar A. Morales, Vladimir V. Verevkin, Vasily A. Bannov, Valentina M. Krasilnikova, Anastasia V. Popova, Eugeni L. Zhilenkov, Johnna K. Garrish, Kathleen M. Schegg, Rebekah Woolsey, David R. Quilici, J. Eric Line, Kelli L. Hiett, Gregory R. Siragusa, Edward A. Svetoch, Bruce S. Seal

**Affiliations:** 1 State Research Center for Applied Microbiology and Biotechnology, Obolensk, Moscow region, Russian Federation; 2 Poultry Microbiology Safety Research Unit, Richard B. Russell Agricultural Research Center, Agricultural Research Service, USDA, Athens, Georgia, United States of America; 3 Nevada Proteomics Center, University of Nevada, Reno, Nevada, United States of America; 4 Danisco/DuPont, Waukesha, Wisconsin, United States of America; University of Ottawa, Canada

## Abstract

*Clostridium perfringens* is a Gram-positive, spore-forming anaerobic bacterium responsible for human food-borne disease as well as non-food-borne human, animal and poultry diseases. Because bacteriophages or their gene products could be applied to control bacterial diseases in a species-specific manner, they are potential important alternatives to antibiotics. Consequently, poultry intestinal material, soil, sewage and poultry processing drainage water were screened for virulent bacteriophages that lysed *C. perfringens*. Two bacteriophages, designated ΦCPV4 and ΦZP2, were isolated in the Moscow Region of the Russian Federation while another closely related virus, named ΦCP7R, was isolated in the southeastern USA. The viruses were identified as members of the order *Caudovirales* in the family *Podoviridae* with short, non-contractile tails of the C1 morphotype. The genomes of the three bacteriophages were 17.972, 18.078 and 18.397 kbp respectively; encoding twenty-six to twenty-eight ORF's with inverted terminal repeats and an average GC content of 34.6%. Structural proteins identified by mass spectrometry in the purified ΦCP7R virion included a pre-neck/appendage with putative lyase activity, major head, tail, connector/upper collar, lower collar and a structural protein with putative lysozyme-peptidase activity. All three podoviral bacteriophage genomes encoded a predicted N-acetylmuramoyl-L-alanine amidase and a putative stage V sporulation protein. Each putative amidase contained a predicted bacterial SH3 domain at the C-terminal end of the protein, presumably involved with binding the *C. perfringens* cell wall. The predicted DNA polymerase type B protein sequences were closely related to other members of the *Podoviridae* including *Bacillus* phage Φ29. Whole-genome comparisons supported this relationship, but also indicated that the Russian and USA viruses may be unique members of the sub-family *Picovirinae*.

## Introduction

*Clostridium perfringens*, a Gram-positive, spore forming, anaerobic bacterium commonly present in the intestines of humans and animals, is classified into one of five types (A, B, C, D, or E) based on toxin production or presence of the toxin-encoding genes [1], [2]. Human clinical symptoms of infection and histolytic pathogenesis are closely associated with the *C. perfringens* enterotoxin (CPE) produced by type A strains. Human food poisoning and gastroenteritis occurs if sufficient numbers of vegetative CPE-positive *C. perfringens* cells are ingested from contaminated food. The vegetative cells may pass from the stomach to the intestinal tract where during sporulation, CPE is released into the intestinal lumen [3]–[5]. CPE-positive type A *C. perfringens* has been implicated in antibiotic-associated and sporadic diarrhea in humans that may also be food-related [6]. In addition to the association of CPE-positive type A strains with food-borne disease in humans, *C. perfringens* type A strains that produce the alpha toxin can cause necrotic enteritis and the subclinical form of infection in poultry [7].

Increased worldwide concerns over antimicrobial resistance (AMR) of zoonotic bacteria potentially circulating among food-producing animals, including poultry [8], has resulted in a heightened public and scientific realization that antibiotic use by humans and in food animals selects for development of AMR among food-borne bacteria [9]. Because of these concerns, sub-therapeutic use of antibiotics as growth promoters was discontinued in the European Union [10] and it is hypothesized that human food-borne and poultry illnesses associated with the bacterium *C. perfringens* may increase as a consequence [7]. Historically, bacteriophages have been used to combat bacterial infections and, recently, there is renewed interest in utilizing live-phages or phage gene products as promising alternative antimicrobials to widely used antibiotics, including the control of food-borne pathogens [11]–[14]. In 2006, the U.S. Food and Drug Administration approved its first mixture of bacteriophages as food additives for use in processing plant spray applications onto ready-to-eat meat and poultry products to protect consumers from *Listeria monocytogenes* [15]. Zimmer et al. [16] isolated two temperate phages (Φ3626 and Φ8533) from lysogenic *C. perfringens* cultures and subsequently expressed a phage-specific enzyme identified as a murein hydrolase [17]. Another endolysin, a muramidase, was cloned and expressed from the episomal ΦSM101 genome [18].

Given the potential use of lytic bacteriophages and/or their lytic enzymes for medical, veterinary and bio-industrial applications, our laboratories have been screening poultry intestinal material, soil, sewage and poultry processing drainage water for obligately lytic bacteriophages from *C. perfringens* [19]. Bacteriophage genomes of viruses previously isolated from broiler chicken offal washes (O) and poultry feces (F) in the USA, designated ΦCP39O and ΦCP26F, respectively, produced clear plaques on host strains and were classified as members of the family *Siphoviridae* in the order *Caudovirales* [20]. Bacteriophages lytic for *C. perfringens* classified in the family *Podoviridae* were isolated from broiler intestinal contents in the Russian Federation, one designated ΦCPV1 [21], and another from raw sewage in the USA, ΦCP24R [22]. Herein we report the molecular characterization of three unique, closely related Russian and US podoviruses that infect *C. perfringens* and present phylogenetic comparisons with members of the sub-family *Picovirinae*.

## Results and Discussion

### Isolation of bacteriophages virulent for *C. perfringens* and virion morphology

**Figure 1:**
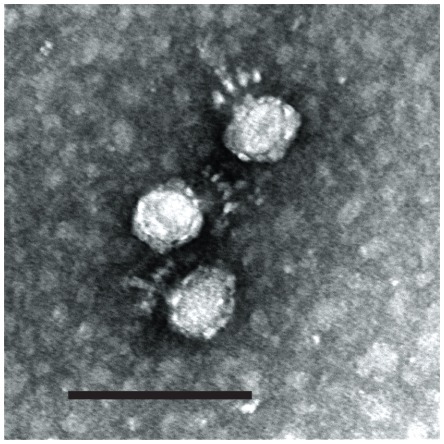
Electron Micrograph of Bacteriophage ΦCP7R. Scale bar represents 100 nm.

Bacteriophages were isolated by screening for lysis of forty-six *C. perfringens* strains [23] utilizing filter sterilized samples obtained from raw sewage or poultry (intestinal material and feces), soil and processing drainage water [20]–[22]. Bacterial viruses capable of lysing strains of the bacterium were identified by spot-testing the filter sterilized concentrated samples and titration of bacteriophages on susceptible *C. perfringens* strains. Individual bacteriophages from the USA were designated by their host strain (i.e., ΦCP7R) while Russian bacteriophages were named numerically (i.e., ΦCpV4 or ΦZP2). Each bacteriophage isolate only replicated in its respective host strain producing clear, 3 mm plaques with no evidence of background bacterial growth (Fig. S1). No other clostridial species assayed for lysis supported replication of these bacteriophages that were routinely obligately lytic for *C. perfringens* with no evidence of lysogeny. The titers of each bacteriophage lysate were approximately 2×10^8^ PFU/ml. Lytic bacteriophage preparations were initially characterized morphologically by electron microscopy (Fig. 1). Two short-tailed bacteriophages isolated from in the Moscow Region of the Russian Federation were designated ΦCPV4 (from poultry waste) and ΦZP2 (from poultry feces), while one virus from the southeastern U.S., isolated from raw sewage, was named ΦCP7R. Each of the bacteriophages were morphologically equivalent with head diameters of approximately 40–42 nm, tail lengths of 35–38 nm, a basal plate with diameters of 30–32 nm that had short protrusions, and collars with a diameter of 27–28 nm. The advanced tail rods did not show thickening in the distal portion. Structurally, all three viruses can be considered members of the order *Caudovirales* in the family *Podoviridae*, characterized by short, non-contractile tails of the C1 morphotype [24].

### Bacteriophage Genome Structures and Predicted Non-structural Protein ORF's

**Figure 2:**
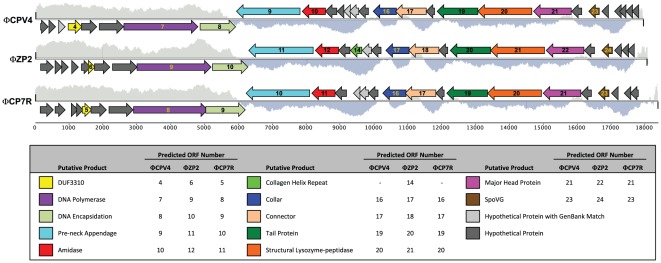
Comparative Genome Maps for Podoviral Bacteriophages Isolated from the Russian Federation and the USA. The %GC plot displays regions (500-bp window) above and below the average GC content. Open reading frames (ORFs) are depicted as arrows in the predicted direction of transcription and identified putative protein domains are listed in the legend with their respective ORF designation.

**Figure 3:**
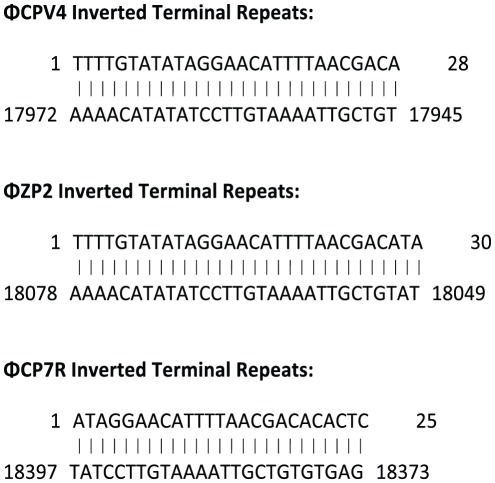
Inverted Terminal Repeat Genomic Nucleotide Sequences for Bacteriophages ΦCPV4, ΦZP2 and ΦCP7R. Inverted terminal repeat (ITR) sequences were identified by whole-genome analysis utilizing the EMBOSS 6.3.1: palindrome program.

The genomes of ΦCPV4, ΦZP2 and ΦCP7R were 17.972, 18.078 and 18.397 kbp, respectively, with an average GC content of 34.6%. Genome sequence identity was 88% for ΦZP2 as compared to genome sequences of ΦCPV4 or ΦCP7R. Sequence identity was 95% between the ΦCPV4 and ΦCP7R genomes even though the bacteriophages were isolated from two disparate geographical regions, Russia and the USA. There were 26, 27 and 28 open reading frames (ORF) identified in the genomes of ΦCP7R, ΦZP2 and ΦCPV4, respectively (Fig. 2; Tables S1, S2, S3). In addition to the previously described morphological characteristics, the identified ORFs revealed protein similarity to previously described podoviral proteins [21], [22] also suggests that the clostridial phages reported herein belong to the *Podoviridae* family. Following alignment of the genomes in Mauve, the principle regions of genomic dissimilarity were in the inverted terminal repeat (ITR) regions at both ends (Fig. S2). The ITRs of the ΦCPV4 genome were 28 nucleotide pairs in length, while the ITRs of the ΦZP2 genome consisted of 30 nucleotide pairs and the ITRs of ΦCP7R were 25 nucleotide pairs (Fig. 3), which are much longer than the ITRs of the Φ29 genome [25]. The small genome sizes and presence of ITRs are characteristics belonging to the subfamily *Picovirinae* [26]. PHACTS analysis [27] predicted that the isolated phages were virulent, which supports our culture observations.

The predicted proteins (Fig. 2; Tables S1, S2, S3) of the *C. perfringens* bacteriophages had demonstrated homology to other known bacteriophage proteins including their DNA polymerases. The predicted Type B polymerases of all three phages were 727 amino acids in length and ΦCPV4 and ΦCP7R were more closely related (99% sequence similarity) than either one compared to the ΦZP2 (96% similar to ΦCPV4 and ΦCP7R). BLAST analyses revealed the three phage proteins were most closely related in sequence to the ΦCPV1 [GenBank ADR30478; 36%] and ΦCP24R [AEW47837; 34%] polymerases with overall similarity of 24% to other DNA polymerases of the *Bacillus* Φ29-family [25], [28]. After the ΦCP24R and ΦCPV1 polymerases, the most closely related polymerases were from the *Bacillus* phages M2 [AAA32368], Nf [ACH57069], GA-1 [CAA65712], B103 [CAA67649] and the *Actinomyces* phage Av-1 [ABR67671]. The predicted DNA polymerase proteins contained a consensus sequence of Dx_2_SSYP rather than the Dx_2_SLYP with the intervening x_2_ being IN rather than VN as found in the Φ29 polymerase motif A. Also, the two Asp (D) residues that participate in metal binding required for catalysis were located in the conserved motif C sequence identical to the Φ29 polymerase [25], [29]. The Φ29-like viruses' genes encoding the terminal protein (TP) have been reported as adjacent to the *pol* gene [25]. Although no putative TP was identified by BLAST analysis of our podoviral genomes, the gene adjacent to the *pol* gene in our podoviruses encoded a protein of a predicted similar structure and physical characteristics to the Φ29 TP. However, a conserved threonine (T) was present in the putative terminal proteins encoded by our phage genomes rather than the serine (S232) in the Φ29 TP [28]. The existence of a terminal protein was further supported by the detection of a protein at the genomic terminal fragments by alteration of their relative mobility following protease and restriction enzyme digestion of ΦCPV4 DNA (Fig. S3).

The ΦCPV4, ΦZP2 and ΦCP7R genomes examined by our laboratories each encoded a putative podovirus DNA encapsidation protein (Fig. 2 and Tables S1, S2, S3). This protein binds to packaging RNA (pRNA) and catalyzes the *in vivo* and *in vitro* genome-encapsidation reaction [30]. These genes encoded a predicted protein of 347 amino acids that was most similar by BLAST to the encapsidation proteins of *Bacillus* phage PZA [AAA88493], *Bacillus* phage Nf [ACH57084] and *Bacillus* phage Φ29 [AAA88348]. This protein functions as a pRNA dependent ATPase providing the energy for encapsidation of the viral DNA into the mature capsid [31], [32]. Two ATP binding motifs occurred in the Φ29 encapsidation protein [33] that were most likely located at residues 34 through 40 for the A motif and residues 267 through 273 for the B motif among the podoviruses reported herein. These motifs were also reported in the encapsidation proteins encoded by the genomes of *Bacillus* phages B103 and GA-1 [25]. Interestingly, the podoviral encapsidation proteins reported during these investigations were more distantly related to DNA encapsidation proteins of our previously reported clostridial ΦCP24R [AEW47838] and ΦCPV1 [ADR30484] relative to the *Bacillus* Φ29 encapsidation protein [YP_002004545].

Other non-structural proteins encoded by the podoviral genomes included a potential stage V sporulation protein G (SpoVG; pfam 04026) that is reportedly essential for sporulation and specific to stage V sporulation in *Bacillus megaterium* and *B. subtilis* [34]. This protein is also involved in the regulation of septum location during cell envelope and outer membrane biogenesis [35]. The SpoVG-like protein encoded by our podoviral genomes were most similar to the regulatory protein SpoVG reported in *Borrelia hermsii* [YP_001884203], *Borrelia turicatae* [YP_945770] and *Stigmatella aurantiaca* [YP_003955432], but were not detected as encoded by the genomes of our previously reported clostridial bacteriophages [19]–[21]. The only viral protein with any sequence similarity was a putative minor capsid protein 4 of the temperate *Streptococcus* phage MM1 [NP_150171]. The *C. perfringens* encoded SpoVG [NP_563407] is composed of only 90 residues compared to our putative podoviral SpoVG proteins of 103 residues and only the first seven N-terminal amino acids were similar between the two proteins. Although the N-terminal sequences were conserved, the functionally essential Proline-63 residue conserved among the SpoVG proteins [35] from *B. subtilis* [CAA44242], *B. hermsii* [YP_001884203] and *C. perfringens* [NP_563407] was missing in the podoviral proteins (F for P in the phage proteins) and it is unknown what role these viral proteins may play in *C. perfringens* biology or during a viral infection.

The three bacteriophages reported encoded a predicted N-acetylmuramoyl-L-alanine amidase (pfam01510), which includes zinc amidases (EC: 3.5.1.28) that cleave the amide bond between N-acetylmuramoyl and L-amino acids in bacterial cell walls (preferentially: D-lactyl-L-Ala). The amidase gene was preceded in the bacteriophage genomes by a gene encoding a predicted pre-neck appendage structural protein (Fig. 2; Tables S1, S2, S3). Similar to the siphoviral *C. perfringens* bacteriophages previously reported from our laboratories, the holin gene is most likely downstream of the lysin gene. This placement is unique to the other clostridial bacteriophages [19], including the podoviruses we recently reported [21], [22]. BLAST analysis of the predicted amidases from ΦZP2, ΦCPV4 and ΦCP7R revealed similarity to a prophage LambdaCh01-like amidase encoded by *C. botulinum* isolates [YP_002863851; YP_001782467; YP_001392135], *C. sporogenes* [ZP_02994334] and *C. tetani* [NP_783826]. The only closely related bacteriophage proteins were to a hypothetical protein BA3_0024 from a *Thalassomonas* phage BA3 [YP_001552293], a PlyM32 from an uncultured bacteriophage [ADF97557], an EBPR siphovirus 4 [AEI71112] and several putative lysozymes from *Vibrio* phages [ADX87661; YP_249586; YP_003347926; ADX87518].

Residues 15 through 130 of the predicted podoviral amidase showed similarity to peptidoglycan recognition proteins (PGRPs), which are pattern recognition receptors that bind and can hydrolyze peptidoglycan (PGNs) of bacterial cell walls [36]. This portion of the protein also contains the substrate binding and putative enzymatically active site, as well as the potential Zn-binding residues. Residues 160 through 225 represented the bacterial Src homology 3 (SH3) domain (pfam08239; superfamily cl02551) that bind to target proteins through sequences containing proline and hydrophobic amino acids. The cell wall targeting-SH3 domains are associated with other hydrolases such as the phage associated cysteine, histidine-dependent amidohydrolase/peptidases (CHAPs), but does not contain the N-terminal ‘F-[IV]-R’ motif common to staphylococcal bacteriophage CHAPs [37], [38]. Interestingly, the C-terminal SH3 portion of the bacteriophage lysins aligned most closely with bacterial CHAP domains, specifically a glycosyl hydrolase, family 25-protein from *C. perfringens* [e.g., ZP_02642156 and seventeen other related sequences]. Since the gene upstream of the amidase encoded a predicted structural protein, synteny with similar bacteriophage genomes suggested the gene downstream from the amidase presumably encoded a holin [19]. However, the small 111 residue gene product does not have a potential transmembrane domain and is predicted to be hydrophilic. The only predicted peptide encoded by the genomes with characteristics of a holin [39], [40], with a single transmembrane domain, is a gene located between the ORF's encoding the phage connector or upper collar protein and a putative tail protein (Fig. 2; Tables S1, S2, S3). The peptide was predicted to have a single transmembrane domain from residues 10 through 32 and also as a potential signal peptide.

### Virion structural proteins

**Figure 4:**
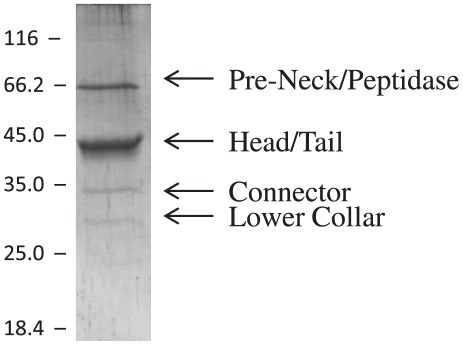
Polyacrylamide Gel Electrophoresis of Virion Proteins from Purified Bacteriophage ΦCP7R. Bacteriophages were purified by isopycnic centrifugation through CsCl followed by electrophoresis in a 10% SDS-PAGE. Size markers are on the left and regions of proteins identified mass spectrometry are on the right side of the figure.

The bacteriophage structural proteins were identified by purifying virions by isopycnic gradient centrifugation through CsCl followed by SDS-PAGE and mass spectrometric analyses of the individual proteins (Fig. 4, Table 1 and Table S4). The principle structural proteins identified included the pre-neck/appendage protein, a putative bacteriophage structural protein, the predicted head protein, a tail protein, the upper collar also known as the connector protein, and a lower collar protein (Fig. 4, Table 1). The virion head of Φ29 consists of a major capsid or head protein that is attached to the tail by a connector or upper collar protein [41]. The head proteins from our podoviral phages were predicted to be 370 residues with a size of 42 kDa, similar to podoviral proteins encoded by viruses previously reported from our laboratories, ΦCP24R [AEW47842] and ΦCPV1 [ADR30492], that belong to a major bacteriophage head protein superfamily [PHA00144]. The proteins were also similar to the head protein of *Actinomyces naeslundii* phage Av-1 [YP_001333662] and *Bacillus* phage Φ29 [YP_002004536]. The connector (portal) or upper collar protein was predicted to be 301 amino acids in length with a size of 34.7 kDa that demonstrated 97% similarity among the three phages. This protein [pfam05352] is the central component of a rotary motor that packages bacteriophage genomic dsDNA into pre-formed proheads [42]. The connector proteins of ΦCPV4, ΦZP2 and ΦCP7R were most similar to those of our previously reported ΦCP24R [AEW47848] and ΦCPV1 [ADR30486] proteins and comparable to the respective proteins of Φ29-like viruses *Bacillus* phages Nf [ACH57078], B103 [NP_690644] and Φ29 [YP_002004539].

**Table 1:** ϕCP7R Virion Proteomics Data Summary as Determined by Mass Spectrometry Analyses.

Protein name	Predicted size (kDa)	Number of unique peptides	Percent coverage
Pre-neck appendage	69.7	10	20.80%
Lysozyme-peptidase	59.2	4	11.30%
Tail protein	46.3	2	6.50%
Major head protein	41.8	18	70.50%
Connector Protein	34.7	6	23.30%
Collar Protein	26.7	15	78.60%

The largest tail protein was a predicted pre-neck/appendage protein that consisted of 624 amino acids for ΦCP7R and ΦCPV4. The predicted pre-neck/appendage for ΦCPZP2 was a 631 residue protein with a relative mobility at approximately 70 kDa and most similar to proteins reported in previously discovered bacteriophages from the USA [AEW47853] and Russia [ADR30483]. The predicted protein contained a putative pectate lyase domain (residues 140 through 350) and these domains are found among bacterial endopygalactorunases involved with cell envelope and outer membrane biogenesis [pfam12708 superfamily protein]. The proteins are encoded in the genomes of *Bacillus* Φ29-like phages [AAA32285] and are appendages attached to the virion neck region [43], [44]. In Φ29 this protein was reported to recognize glucosylated poly-teichoic acids and, thus, is presumably involved with host attachment, cell wall digestion and entry into the cell [44], [45]. The lower collar proteins encoded by our three bacteriophages were 99% similar, consisting of 229 amino acids with a predicted size of 26.7 kDa and belong to the cl10184 superfamily. Again, these proteins from the bacteriophages reported herein were most similar to our previously reported phages ΦCP24R [AEW47853] and ΦCPV1 [ADR30483], but are also related to the lower collar protein of *Bacillus* phage Φ29 [YP_002004540].

The lower portion of the podovirus Φ29 tail below the collar protein consists of two proteins [46]. One tail protein encoded by the three bacteriophages reported was predicted to be 399 amino acids in length (46.2 kDa) with homology to the gp9 of the Φ29-like phages [PHA00380 tail protein; superfamily cl15539]. These proteins were most similar to our previously reported tail proteins of ΦCP24R [AEW47845] and ΦCPV1 [ADR30489] which were much smaller in size relative to homologous tail proteins such as those encoded by *Bacillus* phage GA-1 [NP_073692; 612 aa], *Bacillus* phage B103 [NP_690643; 598 aa] and *Bacillus* phage Φ29 [YP_002004538; 599 aa]. Another structural protein with a predicted size of 529 amino acids (59.2 kDa) was revealed by BLAST analysis to be a multiple-domain protein consisting of a bacteriophage tail domain [PHA00380; PHA00965] (residues 1 through 150), a lysozyme domain [COG3772] (residues 200 through 350), and a peptidase M23 domain [pfam01551] (residues 400 through 500). This tail component was similar by BLAST analysis to proteins reported as a phage structural protein [YP_696017] encoded in the genomes of *C. perfringens* isolates. The predicted protein was also similar to proteins reported in our ΦCP24R [AEW47844] and ΦCPV1 [ADR30490] bacteriophages, as well as the Gp15 protein of *C. perfringens* phage Φ3626 [NP_612844]. The homologous Φ29 gp13 protein is essential for assembly of the Φ29 virion [47] and has two domains related to lysozymes and metallo-endopeptidases [48]. By analogy the 59.2 kDa protein detected in our podoviruses may function similar to the Φ29 gp13 tail-associated, peptidoglycan-degrading enzyme essential for infection.

### Phylogenetics of the Podoviral Bacteriophages from Russia and USA

**Figure 5:**
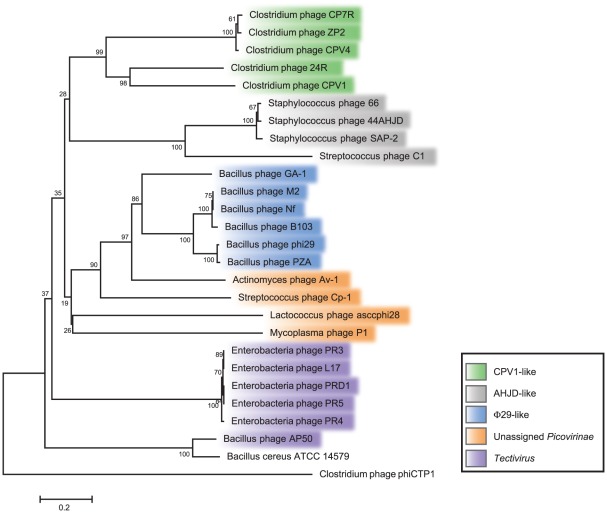
Phylogenetic Relationships of ΦCPV4, ΦZP2 and ΦCP7R among other related Bacteriophages based on the DNA Polymerase Protein. Phylogenetic analysis of polymerase amino acid sequences were completed in MEGA5 utilizing MUSCLE for alignments with two thousand bootstrap replications and the *Clostridium* phage ΦCTP1 polymerase outgroup.

Based on the DNA polymerase predicted amino acid sequences, ΦCPV4, ΦZP2 and ΦCP7R were most closely related to our previously reported *C. perfringens* phages ΦCP24R [AEW47837] and ΦCPV1 [ADR30478] (Figure 5). Beyond the clostridial *Podoviridae*, the other similar bacteriophage polymerases included *Streptococcus* phage C1 [NP_852013], as well as the *Staphylococcus* phages 66 [YP_239478], 44AHJD [NP_817305] and SAP-2 [YP_001491534]. The *Lactococcus* phage accphi28 [YP_001687520] and *Mycoplasma* phage P1 [NP_064636] separated as a cluster with other closely related DNA polymerases of *Streptococcus* phage Cp-1 [NP_044817] and *Actinomyces* phage Av-1 [YP_001333659]. These were most similar to the *Bacillus* phages GA-1 [NP_073685], M2 [AAA32368], Nf [ACH57069], B103 [NP_690635], PZA [AAA88478] and Φ29 [YP_002004529], representing pfam03175type-B DNA polymerases [25]. All of these bacteriophages are considered members of the podoviral sub-family *Picovirinae* and contain type-B DNA polymerases requiring a terminal protein to prime DNA synthesis [49]–[53]. The C-terminal portion, beyond residue 505, of our podoviral predicted polymerase proteins had sequence similarities to members of the *Tectiviridae* such as the enterobacterial phages PRD1 [NP_040682], PR3 [AAX45563], PR4 [AAX45594], PR5 [AAX45625] and L17 [AAX45532] that infect Gram-negative bacterial hosts. These bacteriophages have a lipid membrane underneath the capsid and a protein covalently linked to the 5′-termini of their genomes for priming DNA replication [54]. Other polymerases more distantly related to those reported herein belonged to the *Bacillus cereus* ATCC 14579 [NP_829893] and *Bacillus* phage AP50 [YP_002302517], tentatively also considered members of the *Tectiviridae* [55].

**Figure 6:**
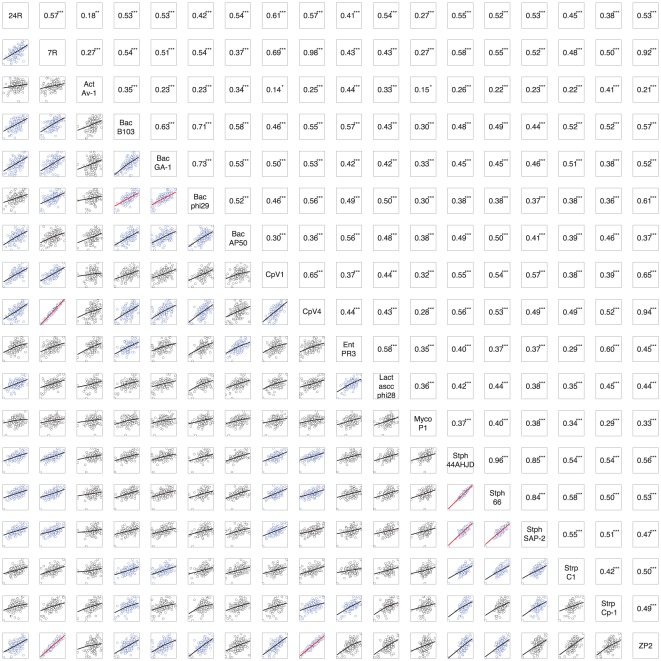
Whole-genome Tetra-nucleotide Frequency Comparisons Relative to Bacteriophages ΦCPV4, ΦZP2 and ΦCP7R. Lower panel shows normalized frequencies of 256 tetranucleotides for each genome, upper panel shows Pearson correlation coefficients. In lower panel, the most closely-related genomes are shown in blue and red. Genome comparisons with correlation coefficients >0.5 are shown with blue points, r-squared values from simple linear regression >0.5 are shown with red lines. Genome names are shown on diagonal axis.

**Figure 7:**
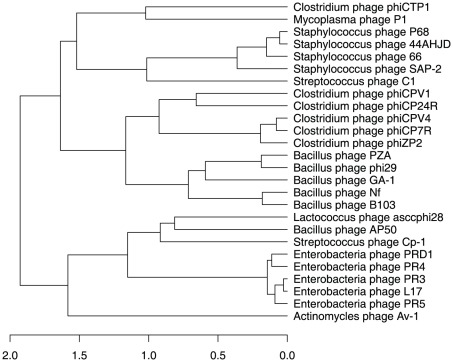
Whole-genome Similarities as Determined by Tetranucleotide Frequencies. Dendrogram is based on a dissimilarity matrix for whole bacteriophage genomes constructed in R as described in the methods section.

Whole-genome analyses of publicly-available picovirus and tectivirus sequences demonstrated that the three phages sequenced here, ΦCPV4, ΦCP7R, and ΦZP2, were most closely related to each other and their closest relatives were two podoviruses (ΦCPV1 and ΦCP24R) previously sequenced by our laboratories (Fig. 6). The use of tetranucleotide frequencies as a metric of whole-genome relatedness has proven to be a valuable tool for comparative genomics [56], [57] and provides a useful alternative to phylogenetic reconstructions based on multiple sequence alignments (MSA). As applied here, the tetranucleotide approach provided an MSA-independent confirmation of the high similarities among ΦCPV4, ΦCP7R, and ΦZP2 with pairwise correlation coefficients of 0.98 (ΦCPV4 vs. ΦCP7R), 0.92 (ΦZP2 vs. ΦCP7R), and 0.94 (ΦZP2 vs. ΦCPV4; Fig. 6). Tetranucleotide comparisons were also consistent with our phylogenetic analyses of the DNA polymerases (Fig. 5), confirming the close relationships among *Staphylococcus* picovirinae, and also the *Bacillus* phages B103, GA-1, and Φ29. The five genomes sequenced by our laboratories belonged to a larger clade containing the *Bacillus* phages, including Φ29 (Fig. 7). The whole-genome phylogeny was slightly different than the polymerase phylogeny in which our phages were most closely related to *Staphylococcus* phages, although this was not strongly supported by bootstrap analysis (Fig. 5). Interestingly, queries of the genomes shown in Fig. 7 for putative paralogs showed only three potential gene duplications; the head proteins of ΦCP24R (ORFs 10 and 11) which had been previously noted [22], hypothetical proteins of ΦZP2 (ORFs 13 and 14) and putative DNA polymerases in ΦCPV1 (ORFs 5 and 6).

**Figure 8:**
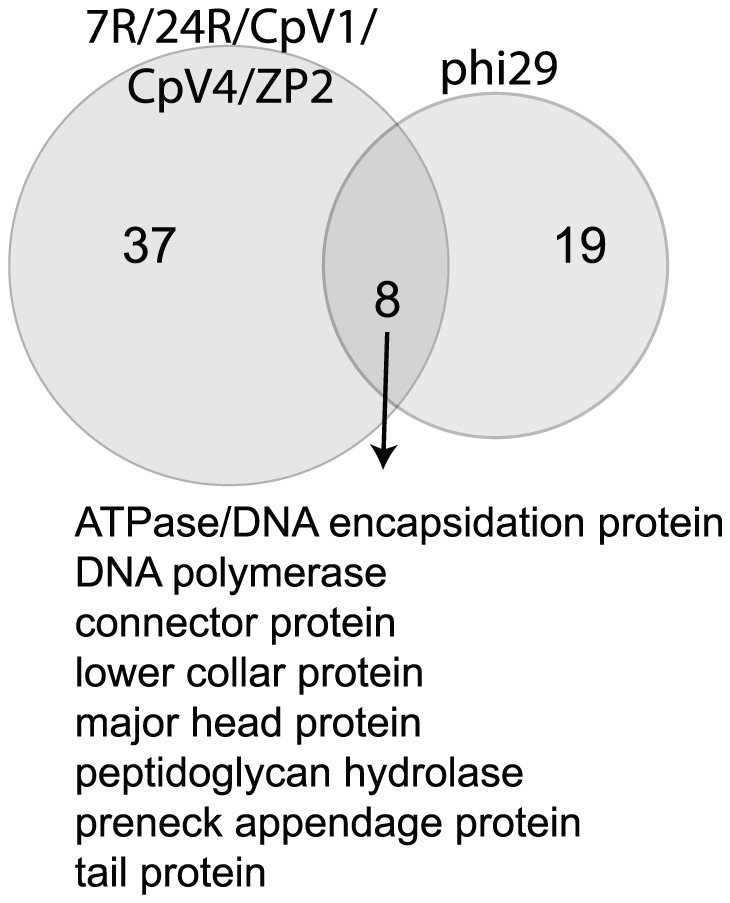
Shared and Unique Genes present in Bacillus phage Φ29 versus Bacteriophages ΦCPV4, ΦZP2 and ΦCP7R. Gene clustering was determined by uclust and blastp as described in the methods section.

As a standard reference genome with important commercial applications [58], we were interested to determine proteins shared by Φ29 and the ‘pan-genome’ of ΦCP7R, ΦCP24R, ΦCPV1, ΦCPV4 and ΦZP2 (Fig. 8). Genes encoding for eight major bacteriophage proteins were shared by these six genomes that consisted of a DNA encapsidation protein, DNA polymerase, connector protein, lower collar protein, major head protein, peptidoglycan hydrolase, preneck appendage protein and a tail protein. These are all proteins encoded by the Φ29 family of lytic bacteriophages that have similar genome structures with a terminal protein and encode a Type B DNA polymerase typical of the *Picovirinae* [25].

### Concluding Remarks

Bacteriophages constitute the largest number of biological entities found on earth [59] and there is resurgent interest for utilizing either bacteriophages [60], [61], or their gene products [12], [14], to control bacterial infections that plague humans, animals and plants. Consequently, we have conducted collaborative research between the Russian Federation and USA to search for bacteriophages that clearly lyse the bacterium *Clostridium perfringens*. This pathogen causes a plethora of diseases in humans and animals that are both medically and agriculturally important [2]. Previously we reported on bacteriophages that were both members of the *Siphoviridae* [19], [20] and *Podoviridae* [21], [22] that met our criteria for producing clear plaques on the bacterium *C. perfringens*. We have now identified three more highly-related bacteriophages from disparate geographical regions virulent for the host *C. perfringens* and demonstrate their inclusion as unique members of the Φ29-like *Picovirinae*.

## Materials and Methods

### Bacterial hosts, bacteriophage isolation and propagation

*C. perfringens* isolates utilized as hosts for propagation of bacteriophages were cultured in brain heart infusion (BHI) broth or on agar (Remel, Lenexa, KS) and characterized by standard methods including 16S rRNA-DNA sequence analysis [23], [62]. Offal washes (O) and feces (F), obtained at chicken processing facilities, or raw sewage samples (R) were collected following verbal consent from poultry processing facility management or personnel at the Athens Clarke County, GA, USA sewage treatment facility and under similar circumstances in the Russian Federation. Samples were clarified by low-speed centrifugation (5,000× g for 20 min. at 5C) followed by filtration of the supernatant through cheesecloth, then by filtration through 0.45 µm bottle filters (Corning Inc., Corning, NY). Bacteriophages from the USA were isolated on their respective host strain (Cp7), while Russian bacteriophages were isolated on *C. perfringens* strains ATCC3624 and strain 46 (provided by the Tarasevich State Institute of Standardization and Control of Biomedical Preparations, Russia). Bacterial viruses producing clear plaques on individual strains of *C. perfringens* were identified by spot-testing and titration on each strain-specific host [20], [21], [63]. Briefly, 10 µl of filtrate was spotted onto lawns of *C. perfringens* and incubated for 16 hours, followed by visualization for areas of clearance indicating lysis of the bacterium. Several clostridial species including *C. absonum*, *C. acetobutylicum*, *C. beijerinckii*, *C. novyi*, *C. rubrum*, *C. sordelli*, *C. sporogenes*, *C. tetani*, and *C. tetanomorphum* [62] were spot-tested for lytic activity. The *C. perfringens*-specific bacteriophages were propagated by plating with low-melt agar using the *C. perfringens* host bacterium cultured at 37C in the Anaero Pack™ (Mitsubishi Gas Chemical Co., Japan) system with AnaeroGen (OXOID Ltd., Basingstoke, England) sachets [20], [23]. The bacteriophage isolates were subjected to three rounds of plaque purification and suspended in TMGS (10 mM Tris, pH 8, 10 mM Mg++, 0.55% NaCl, 0.1% gelatin) at an average titer of 2×10^8^ pfu/ml. Bacteriophages ΦCPV4, ΦZP2 and ΦCP7R were propagated utilizing a plate lysis method [64] under anaerobic conditions.

### Purification of bacteriophages, genomic DNA purification and electron microscopy

Following plate lysis [64] in anaerobic chambers, bacteriophage genomic DNA was purified using the Qiagen™ Lambda Phage DNA isolation protocol. Additionally, bacteriophages were purified from plate lysates by centrifugation at 2,000× g for 20 minutes to remove bacterial debris and low-melt agarose. The clarified supernatant was centrifuged at 103,800× g for 90 minutes followed by suspension of the phage pellet in 1 ml TBS (20 mM Tris, 500 mM NaCl at pH 7.5) followed by purification using cesium chloride equilibrium gradient centrifugation [65]. The bacteriophage bands were extracted from the gradient, diluted in TBS and concentrated by centrifugation at 105,000× g in a Beckman™ JS 24.15 rotor for 90 minutes followed by suspension in TBS. Purity of bacteriophages was examined by electron microscopy [66]. The bacteriophage pellets were also subjected to proteinase K (20 ug/ml) digestion in the presence of 0.1% sarcosyl and 0.2 M EDTA followed by phenol-chloroform extraction and ethanol precipitation to obtain genomic DNA [65].

### Molecular cloning, sequencing, annotation of genomic DNA and phylogenetic analyses

Following purification of bacteriophage genomic DNA, the nucleic acid was subjected to spectrophotometer readings at 260/280 nm and restriction enzyme digestion followed by agarose gel electrophoresis [65]. Sequencing of the bacteriophage genomes was completed by MWG Biotech, Inc High Point, NC USA and pyrosequencing [67]. For Sanger sequencing, phage DNA was sheared using a nebulizer, blunt-end repaired and dephosphorylated [65]. DNA fragments of desired size (1 to 4 kb) were ligated into pSmart (Lucigen™) for propagation in *E. coli* following transformation. Clones were sequenced such that approximately 14-fold redundancy was obtained for the genome that included primer-walking to fill gaps [68]. Molecular cloning was also completed using the restriction enzymes *Hin*dIII, *Eco*RI, *Eco*RV, *Alu*I and *Cla*I (New England Biolabs, Ipswich, MA) to cleave phage DNA followed by treatment with *Taq* polymerase [69] and cloning [70] into the TOPO TA vector (Invitrogen™, Carlsbad, CA). Additionally, end-repair and G-tailing was completed for cloning restriction enzyme fragments into pSmart vectors (Lucigen™, Middleton, WI) for nucleotide sequencing. Double stranded nucleotide sequencing reactions using fluorescent labeled dideoxynucleotide terminators were also completed and sequences determined using an automated sequencer (Applied Biosystems Inc., Foster City, CA) [71].

Nucleotide sequence assembly, editing, analysis, prediction of amino acid sequences and alignments were conducted using the MacVector 7.2™ (Accelrys, San Diego, CA) and DNASTAR™ (Madison, WI) software. The European Molecular Biology Open Software Site was accessed to identify the inverted terminal repeats using EMBOSS 6.3.1: palindrome [72]. Protein-encoding genes (ORFs) were predicted using GeneMark.hmm for prokaryotes version 2.4 (http://opal.biology.gatech.edu/GeneMark) [73] and SoftBerry FGENE SB (http://linux1.softberry.com/berry.phtml; Mount Kisco, NY, USA) programs. Final genome sequences were also submitted to the IMG/ER pipeline for gene predictions and initial annotation [74]. The computational approach Phage Classification Tool Set (PHACTS) was developed to classify phages as to whether the lifestyle of a phage, described by its proteome, is virulent or temperate by using the known phage genomes in the PHANTOME database. PHACTS utilizes known genomes to find similarities in the unknown phage genome and was used to predict whether our bacteriophages were virulent or temperate [27]. Predicted ORFs were searched for similarity to proteins in databases by BLAST analyses [75] at the NCBI website (http://www.ncbi.nlm.nih.gov) as well as the conserved domain database [76] algorithms with NCBI accession numbers and domain designations reported in the results for similar proteins. Putative promoters were analyzed by using Martin Reese's neural network prediction program at http://www.fruitfly.org/seq_tools/promoter.html and BPROM (Softberry, Inc., Mount Kisco, NY, USA) at its website http://linux1.softberry.com/berry.phtml. Potential transcriptional terminators were assessed using the software programs TransTerm at the Nano+Bio-Center (http://nbc3.biologie.uni-kl.de) and FindTerm (Softberry, Inc., Mount Kisco, NY, USA) at the web site http://linux1.softberry.com/berry.phtml. The amino acid sequences of the phage ORF gene products were analyzed for helical transmembrane domains by using the prediction program TMHMM version 2.0 [77] at the website http://www.cbs.dtu.dk and by Dense Alignment Surface method [78] at http://www.sbc.su.se/ ~melen/TMHMMfix/ and http://phobius.sbc.su.se/.

Multiple genome alignments were generated by Mauve software to provide for comparative analyses of multiple bacteriophage genomes [79], [80]. Phylogenetic analyses of polymerase amino acid sequences were completed in MEGA5 [81] utilizing MUSCLE for alignments with two thousand bootstrap replications [82] and an outgroup [83], [84]. Tetra-nucleotide distributions for clostridial phage genomes and correlation coefficients between genomes were calculated with TETRA [56]. Correlation coefficients were transformed to a dissimilarity matrix for tree construction using the hierarchical clustering algorithm hclust in R [85], which was also used to generate dendrograms and visualize tetra-nucleotide distributions. Additional whole-genome comparisons were performed by reciprocal blastp with an e-value cutoff of 0.001 to identify core and accessory proteins between the clostridial phages sequenced here and several reference phage genomes. The number of genes shared among the five clostridial phages sequenced by our laboratories was determined by UCLUST [86] which identifies similarity clusters based on all-versus-all sequence comparisons. Blastp with an evalue cutoff of 0.001 was used to confirm genes shared between the clostridial phages sequenced here and the reference phage genome of Φ29.

### Preparation of purified virions for gel electrophoresis and identification of purified bacteriophage proteins by mass spectrometry

Following purification of bacteriophages by isopycnic centrifugation in CsCl the bacteriophage bands were dialyzed against TBS and the dialyzed bacteriophage preparation was centrifuged to pellet the viruses [20]. The virus pellet was suspended in electrophoresis buffer followed by SDS-PAGE [87]. Selected bands were digested using a previously described protocol with some modifications [88], [89]. Samples were washed twice with 25 mM ammonium bicarbonate (ABC) and 100% acetonitrile (ACN), reduced and alkylated using 10 mM dithiothreitol and 100 mM iodoacetamide then incubated with 75 ng sequencing grade modified porcine trypsin (Promega, Fitchburg WI) in 25 mM ABC overnight at 37C. Peptides were first separated by a Paradigm Multi-Dimensional Liquid Chromatography (MDLC) instrument (Michrom Bioresources Inc., Auburn, CA) with a Magic C18AQ 3 µ 200 Å (0.2×50 mm) column, (Michrom Bioresources Inc.) using a ZORBAX 300SB-C18 5 µ (5×0.3 mm) trap (Agilent Technologies, Santa Clara, CA). The flow rate was 4 µl/min and the solvent gradient was from 5% B (5 min) to 45% B over 90 min, then 80% B (1 min). Solvent A was 0.1% aqueous formic acid and solvent B contained 0.1% formic acid in ACN. Eluted peptides were analyzed using a LTQ-Orbitrap XL (ThermoElectron, Bremen Germany) equipped with a Captive Spray source (Michrom Bioresources Inc.) using Xcalibur v2.0.7. The MS was operated in data-dependent mode switching between Orbitrap-MS and LTQ-MS/MS. Full scan MS spectra (*m*/*z* 300–1800) were acquired in the positive ion mode with resolution of 60,000 in profile mode. The five most intense data-dependent peaks were subjected to MS/MS using collision-induced dissociation with a minimum signal of 2,000 and isolation width of 3.0 with normalized 35.0 collision energy. Ions already selected were dynamic excluded for 30 seconds after a repeat count of 2 with a repeat duration of 10 seconds. A reject mass list was used which included known background ions and trypsin fragments.

The MS/MS data were extracted using Sorcerer v3.5 (Sage-N Research, Milpitas CA.). Charge state deconvolution and deisotoping were not performed. All MS/MS samples were analyzed using Sequest (Thermo Fisher Scientific, San Jose, CA, version v.27, rev. 11). The search was performed using a combined version of all *Clostridium* spp. entries out of NCBI and in-house bacteriophage sequences (data from these investigations) with a random, concatenated decoy database added (including 529,702 entries) assuming the digestion enzyme trypsin. Sequest was searched with a fragment ion mass tolerance of 1.00 Da and a parent ion tolerance of 10 ppm. Iodoacetamide derivative of cysteine was specified in Sequest as a fixed modification, oxidation of methionine was specified in Sequest as variable. Scaffold (version Scaffold_3.3.1, Proteome Software Inc., Portland, OR) was used to validate MS/MS based peptide and protein identifications. Peptide identifications were accepted if they could be established at greater than 95.0% probability as specified by the PeptideProphet algorithm [90]. Protein identifications were accepted if they could be established at greater than 95.0% probability and contained at least 2 identified peptides. Protein probabilities were assigned using the Protein Prophet algorithm [91]. Proteins that contained similar peptides and could not be differentiated based on MS/MS analysis alone were grouped to satisfy the principles of parsimony. The False Discovery Rate (FDR) was calculated by Scaffold using an empirical method by Kall et al. [92].

## Supporting Information

Figure S1 **Representative Plaque Morphology.** Bacteriophage ΦCP7R was propagated in the host *Clostridium perfringens* Cp7 for titration and plaques were photographed with an AlphaImagerHP.
(EPS)

Figure S2 **Whole Genome similarity Comparison for Bacteriophages ΦCPV4, ΦZP2 and ΦCP7R.** The bacteriophage genomes were aligned in Mauve for comparative genomics to determine overall sequence similarity and if any rearrangement, segmental duplication, gain, or loss created a mosaic pattern of homology.
(EPS)

Figure S3 **Restriction Endonuclease Maps for the Genomes of Bacteriophages ΦCPV4, ΦZP2 and ΦCP7R and Terminal Protein Detection.** A) *Eco*RV and *Eco*91I maps for all three bacteriophages based on genome nucleotide sequences. B) Electropherogram of ΦCPV4 genomic fragments following digestion with protease K and *Eco*RV (lane 1) and *Eco*RV without protease digestion (lane 2). Arrows indicate terminal genome fragments.
(EPS)

Table S1 **Open Reading Frames for the ΦCPV4 Genome.**
(XLS)

Table S2 **Open Reading Frames for the ΦZP2 Genome.**
(XLS)

Table S3 **Open Reading Frames for the ΦCP7R Genome.**
(XLS)

Table S4 **Purified Bacteriophage Protein Mass Spectrometry Proteomics Data.**
(XLSX)
